# Biomarker-Measured Unhealthy Alcohol Use in Relation to CD4 Count Among Individuals Starting ART in Sub-Saharan Africa

**DOI:** 10.1007/s10461-018-2364-2

**Published:** 2018-12-17

**Authors:** Jessica F. Magidson, Robin Fatch, Catherine Orrell, Gideon Amanyire, Jessica E. Haberer, Judith A. Hahn, the META team, Jessica Haberer, Catherine Orrell, Norma Ware, Mwebesa Bosco Bwana, Asiimwe Stephen, Amanyire Gideon, Tumwesigye Elioda, David Bangsberg, Alex Tsai, Mark Siedner, Lynn Matthews, Ingrid Katz, Monique Wyatt, Nomakhaya April, Alienah Mpahleni, Vivie Situlo, Speech Mzamo, Nomsa Ngwenya, Khosi Tshangela Regina Panda, Teboho Linda, Christine Atwiine, Sheila Moonight, Edna Tindimwebwa, Nicholas Mugisha, Peace Atwogeire, Vian Namana, Catherine Kyampaire, Gabriel Nuwagaba, Annet Kembabazi, Stephen Mugisha, Victoria Nanfuka, Anna Cross, Nicky Kelly, Daphne Moralie, Kate Bell, Nicholas Musinguzi, Dolphina Cogill, Justus Ashaba, Zoleka Xapa, Mathias Orimwesiga, Elly Tuhanamagyezi, Catherine Kyampaire, Don Bosco Mpanga, Leonia Kyarisima, Simone Kigozi, Edgar October, Silver Mugisha, Ibrahim Kiviiri

**Affiliations:** 1 Department of Psychology, University of Maryland, 1147B Biology-Psychology, College Park, MD 20742 USA; 2 Department of Medicine, University of California San Francisco (UCSF), San Francisco, CA USA; 3 Desmond Tutu HIV Centre, Department of Medicine and Institute of Infectious Disease and Molecular Medicine, University of Cape Town, Cape Town, South Africa; 4 Joint AIDS Program, Makerere University, Kampala, Uganda; 5 Massachusetts General Hospital and Harvard Medical School, Boston, MA USA

**Keywords:** Alcohol use, Biomarker, HIV treatment, Sub-Saharan Africa, Uso de alcohol, Marcador biologico, Tratamiento VIH, Africa sub-Sahariana

## Abstract

Individuals are initiating antiretroviral therapy (ART) at earlier HIV disease stages. Unhealthy alcohol use is a known barrier to successful HIV treatment outcomes, yet it is unclear whether the problem varies by disease stage. We measured alcohol use with an objective biomarker (phosphatidylethanol [PEth]), comparing individuals (*n *= 401) with early (CD4 > 350 cells/mL, WHO Stage 1) versus late (CD4 < 200 cells/mL) ART initiation in HIV care in Uganda and South Africa (SA). We examined the association between CD4 count and biomarker results using multivariable regression modeling, and compared PEth results to self-report to assess underreporting. Overall, 32.2% (*n *= 129) had unhealthy alcohol use (PEth ≥ 50 ng/ml). Early ART initiation was significantly associated with unhealthy alcohol use in Uganda (AOR 2.65; 95% CI: 1.05–6.72), but not SA (AOR 1.00; 95% CI: 0.46–2.17). In Uganda, 23.2% underreported unhealthy alcohol use versus 11.6% in SA (*χ*^2^ = 9.30; *p *< 0.01). Addressing unhealthy alcohol use is important as patients initiate ART earlier, yet challenging due to underreporting.

## Resumen

Los pacientes con VIH estan iniciando terapia antiretroviral (TAR) en estadio temprano de la enfermedad. El consumo perjudicial de alcohol es una barrera conocida para el tratamiento efectivo de el VIH, aunque todavia no esta claro si el problema varia dependiendo del estadio de la enfermedad. Evaluamos los niveles de alcohol con el marcador biologico especifico Fosfatidil-etanol (PEth), comparando individuos (*n *= 401) al inicio (CD4 > 350 celulas/mL, OMS estadio 1) versus tardio (CD4 < 200 celulas/mL) de la TAR en Uganda y Africa del el Sur (AS). La asociacion entre el conteo de CD4 y los resultados de PEth se realizo mediante un analisis de regresion multivariable y ademas comparamos los resultados del PEth con el reporte voluntario con el fin de medir la tasa de bajo reporte. En general, 32.2% (*n *= 129) tenian consumo perjudicial de alcohol (PEth ≥ 50 ng/mL). En Uganda el inicio temprano de la TAR esta significativamente ligado al consumo perjudicial de alcohol (AOR = 2.65; 95% CI: 1.05-6.72) a diferencia de Africa de el Sur (AOR = 1.00; 95% CI: 0.46-2.17). En Uganda se detecto un 23.2% de bajo reporte de consumo frente a un 11.6% en AS (χ^2^ = 9.30: *p* < 0.01). Identificar el uso perjudical de alcohol es importante debido a que los pacientes inician tempranamente la TAR, esto se dificulta debido a la baja tasa de reporte.

## Introduction

New universal access initiatives may bring 23 million people on antiretroviral therapy (ART) at healthier stages than previously, when ART was given only to those with low CD4 cell counts [1]. In areas with generalized HIV epidemics, such as sub-Saharan Africa, one of the primary barriers to successful HIV treatment and secondary prevention is unhealthy alcohol use [2–4]. Unhealthy alcohol use is a consistent risk factor for HIV acquisition, as well as poorer HIV treatment outcomes due to ART nonadherence [5–9]. As individuals will be initiating ART earlier than ever before, the extent to which alcohol use is a problem among those starting ART at high CD4 cell counts is unknown. Poor physical health status and worsening of one’s health have both been associated with lower alcohol consumption among individuals with HIV following ART initiation [10] as well as at the onset of other chronic diseases [11, 12]. As such, as individuals are initiating ART when they are more likely to be asymptomatic and with fewer physical symptoms, unhealthy alcohol use at ART initiation may be a barrier to ART adherence and successful treatment outcomes.

Yet, efforts to recognize and address unhealthy alcohol use may be limited by low detection rates using self-report screening methods [13–15]. Patients, including those in sub-Saharan Africa, have been shown to be reluctant to disclose alcohol use to their health care providers due to factors related to stigma, social desirability biases, and fear of being denied ART [8, 16]. Additionally, lack of standardized alcohol content and serving size may also contribute to inaccurate consumption reports [17].

Phosphatidylethanol (PEth), a phospholipid metabolite of ethanol, offers an objective measure of alcohol use. PEth is formed only in the presence of alcohol use and is thus one of the most sensitive and specific biomarkers for alcohol intake over the prior two to three weeks [18]. Detecting PEth at the time of ART initiation offers an objective measure of alcohol use early on among individuals initiating ART. Further, although prior research has identified high rates of underreporting of alcohol consumption using PEth testing in those in HIV care in Uganda [8, 17, 19], it is unknown whether this is generalizable to other sub-Saharan African countries. This question is particularly relevant in South Africa, where there is a high prevalence of HIV and alcohol use, as well as other illicit substance use, which is often stigmatized [20, 21].

As part of a recent study [3] that followed individuals initiating ART at high CD4 count (CD4 > 350 cells/μL and asymptomatic) versus low CD4 counts (CD4 < 200 cells/μL) in routine care in southwestern Uganda and Cape Town, South Africa, we conducted an analysis to (1) compare the prevalence of biomarker-measured unhealthy alcohol use (PEth ≥ 50 ng/mL) among individuals initiating ART early versus late in routine care in Uganda and South Africa; and (2) determine whether differences persisted after adjusting for relevant covariates. Secondary aims were to assess the prevalence of underreporting unhealthy alcohol use (i.e., PEth ≥ 50 ng/mL without self-reported unhealthy alcohol use), and whether rates of underreporting differed by level of CD4 count and site after adjusting for relevant covariates, including perceived health status. Given prior research demonstrating that worse physical health status is associated with reduced alcohol consumption in HIV [10] as well as other chronic conditions [11, 12], we hypothesized that individuals who are starting ART when they are asymptomatic and with high CD4 counts would have higher alcohol use compared to individuals initiating ART later with lower CD4 counts, and that underreporting would be higher in Uganda compared to South Africa.

## Methods

### Study Procedures

Individuals were recruited using convenience sampling as they presented for HIV care. Inclusion criteria for the parent study included: (1) ART-naïve and initiating ART within one  month of enrollment date; (2) either CD4 count > 350 cells/μL and asymptomatic (clinically categorized based on WHO Stage 1 criteria) or CD4 count < 200 cells/μL at any WHO stage; (3) at least 18 years of age; (4) living within 60 km of the clinic; (5) intention to stay in the area for the next year; (6) if pregnant, no more than 34 weeks gestation; and (7) able to provide informed consent. Participants were excluded if the met any of the following criteria: (1) intermediate stage disease at enrollment (i.e., CD4 200–349); or (2) if pregnant, late stage disease (CD4 < 200). All participants initiated treatment within approximately one week of the assessment (median = 1; range 1–8). Please see Haberer et al. for additional detail about the parent study [3].

We randomly selected 432 stored dried blood spots (DBS) from the baseline assessments of the parent study (*n *= 692) for PEth testing, which included 108 from each enrollment group, at each site (early and late initiators, pregnant women excluded). We compared those with completed PEth testing and self-reported alcohol use (AUDIT-C) data (*n *= 401) to those without (*n *= 291). Missing data was due to no DBS sample available (*n *= 5), missing AUDIT-C data (*n *= 26), or not selected for PEth testing (*n *= 260). A higher percentage of female participants were included compared to male participants (61% vs. 53%, *p *= 0.02), and study enrollment date was significantly later for those not included (*p *< 0.01). There were no other significant differences for any variable included in the study multivariable analyses (see below; all *p*’s > 0.05).

This study was approved by Partners Healthcare/Massachusetts General Hospital, Mbarara University of Science and Technology, the Uganda National Council for Science and Technology, the University of Cape Town, and the Western Cape province in South Africa. Procedures were conducted in accordance with the Helsinki Declaration of 1975, as revised in 2000. Informed consent was obtained from all individual participants included in the study.

### Assessments

The parent study collected a comprehensive battery of sociobehavioral measures via structured interviewer-administered interview at baseline, six months, and 12 months. The current analysis focused on the study assessments described below, conducted at baseline.

*AUDIT-C* Self-reported alcohol use was measured using the Alcohol Use Disorder Identification Test—Consumption (AUDIT-C) [22]. The AUDIT-C assesses alcohol use and related problems in the prior three months; unhealthy alcohol use via self-report was defined as an AUDIT-C score ≥ 3 for women and ≥ 4 for men.

*Biomarker (PEth)* We used PEth, a biomarker of recent alcohol use, to objectively measure alcohol use. PEth remains in the blood for approximately 21 days after alcohol consumption and represents a useful objective measure of alcohol use [8, 15]. In prior studies, PEth has demonstrated superior sensitivity (88%) and specificity (89%) for any alcohol use over 21 days among individuals living with HIV/AIDS in Uganda, and it has greater diagnostic specificity and sensitivity than other frequently used alcohol biomarkers, such as carbohydrate-deficient transferrin (CDT) and gamma-glutamyl transpeptidase (GGT) [18]. PEth is formed only in the presence of alcohol use, and thus is highly specific. Further, PEth’s test characteristics have been shown to not vary with CD4 count, ART status, and age [15].

PEth was tested at the United States Drug Testing Laboratories, Inc. (USDTL) using liquid chromatography-tandem mass spectrometry (LC–MS/MS) after methanol was extracted from dried blood spots (DBS) [23]. The lower level of quantification was 8 ng/mL. Unhealthy alcohol use via PEth was defined as a PEth result ≥ 50 ng/mL, a cutoff which was highly sensitive (93%) and reasonably specific (83%) for detecting daily drinking of at least two drinks per day on average in a study of 222 patients with liver disease (S. Stewart, personal communication). We used PEth as the marker for unhealthy alcohol use due to findings that under reporting varied significantly by site (see Results).

### Underreporting Alcohol Use

Participants who had a PEth result ≥ 50 ng/mL but who did not meet the AUDIT-C thresholds for unhealthy alcohol use were defined as underreporters of unhealthy alcohol use.

### Health Status

*CD4 count* CD4 point-of-care testing was conducted at enrollment using Alere Pima™. The “early” CD4 group was defined as CD4 count > 350 cells/μL with asymptomatic HIV disease (clinically categorized based upon WHO Stage 1 criteria), and the “late” CD4 group was defined as CD4 count < 200 cells/μL (any WHO stage).

*Perceived physical and mental health status* Self-reported health status was measured using the MOS-HIV, a measure of health-related quality of life that has been validated in sub-Saharan Africa (normed with 50 as average health in the US population) [24]. The physical and mental health summary scores (PHS and MHS, respectively), were used to measure perceived physical health-related functioning (PHS) and well-being (MHS) in this study. The PHS and MHS scales have been validated against clinical measures in HIV care in Uganda [25]; low PHS and MHS scores have been shown to correspond with low CD4 count and distinguish between stages of HIV infection, with individuals who are asymptomatic (WHO stage 1) scoring significantly higher on the PHS and MHS than individuals who were symptomatic at later disease stages [25].

*TB status* was assessed based on self-reported use of TB medication at the time of enrollment.

### Covariates

We considered the following as covariates because they have been shown to be associated with alcohol use and underreporting, including demographic characteristics, structural barriers to care, stigma, and depression [26, 27].

*Demographic characteristics* Demographic characteristics included participant gender, age, education, and economic status. Age was categorized as follows: ≤ 25, 26–35, 36–45, > 45 years. Education was categorized as follows: none, primary school (P1–P6 in Uganda/G1–G7 in South Africa), high school (P7–S6 in Uganda/G8–G12 in South Africa), and more than high school (> S6 in Uganda/> G12 in South Africa). We used principal components analysis to create a household asset index from measures of housing quality and available energy sources [28, 29]; participants were grouped into the following categories: low (bottom 40%), middle (middle 40%), high (top 20%).

*Stigma* was measured using a 15-item abridged version of the Berger HIV Stigma scale [30], which included two subscales: HIV disclosure concerns and perceived negative attitudes. This measure has demonstrated strong internal consistency, reliability, and validity [30].

*Structural barriers to care* was assessed using a 13-item structural barriers to clinic attendance subscale of a previously validated measure in South Africa of structural barriers to care and ART adherence among individuals on ART [31]. This subscale has demonstrated excellent internal consistency in prior work in South Africa [31].

*Depression* We used a modified 12-item version of the Hopkins Symptom Checklist to measure depression that excluded somatic measures of depression [32, 33].

### Statistical Analysis

All analyses were limited to study participants with PEth testing and self-reported alcohol use (AUDIT-C) data available (*n *= 401). To describe the study participants, we calculated frequency distributions for categorical variables, and medians and interquartile ranges (IQRs), means (*M*) and standard deviations (*SD*) for continuous variables, overall and stratified by CD4 group and study site. To assess associations with PEth-measured unhealthy alcohol use and underreported unhealthy alcohol use, we conducted bivariate analysis and multivariable logistic regression models, stratified by site. We stratified the analyses by site given socio-economic/cultural differences at the sites, and the different prevalence of underreporting at the two study sites. The following variables were selected a priori for inclusion in multivariable models: CD4 group, gender, age, household asset index, self-reported physical and mental health status, the Hopkins depression score, disclosure concerns stigma score, perceived negative attitudes stigma score, months since HIV diagnosis, study enrollment date, and structural barriers to care. We also conducted a posthoc analysis to test whether the interaction between one’s CD4 group (high vs. low) and perceived physical health status (PHS; continuous measure) was associated with unhealthy drinking and underreporting above and beyond perceived health status and CD4 group independently.

## Results

### Demographic and Clinical Characteristics

Of the total sample (*n *= 401), 63.6% were female, median age was 32 years (IQR 27–40), and the median annual income was $370 (US dollars; IQR 0–1481). Half (*n *= 201) were in the low CD4 group (< 200 cells/μL and any WHO stage) versus high CD4 count and asymptomatic (> 350 cells/μL and WHO Stage 1; *n *= 200). Approximately half (53%; *n *= 211) were recruited in Uganda (vs. South Africa; *n *= 190). Median CD4 count in the high CD4 group was 434.5 cells/μL (IQR 392–481), and median CD4 count in the low CD4 group was 121 cells/μL (IQR 55–160). Regarding perceived health status, individuals who were in the high CD4 group and asymptomatic (based on WHO Stage 1 criteria) had a mean PHS score of 42.3 (*SD *= 6.8) compared to 38.7 (*SD *= 6.5) in the low CD4 group. Table 1 presents other demographic and clinical characteristics by CD4 count group and site.

**Table 1:** Demographic, clinical, and alcohol use characteristics of persons starting ART by CD4 count and study site

	Overall (n = 401)	High CD4 group (CD4 > 350) (n = 200)	Low CD4 group (CD4 < 200) (n = 201)	Uganda (n = 211)	South Africa (n = 190)
	N (%)	N (%)	N (%)	N (%)	N (%)
Demographics					
Gender					
Male	146 (36.4)	54 (27.0)	92 (45.8)	84 (39.8)	62 (32.6)
Female	255 (63.6)	146 (73.0)	109 (54.2)	127 (60.2)	128 (67.4)
Age [median (IQR)] (n = 400)	32 (27–40)	31 (25–40)	33 (28–40)	30 (25–37)	34 (29–42)
≤ 25	82 (20.5)	55 (27.5)	27 (13.5)	56 (26.5)	26 (13.8)
26–35	175 (43.8)	78 (39.0)	97 (48.5)	92 (43.6)	83 (43.9)
36–45	86 (21.5)	37 (18.5)	49 (24.5)	39 (18.5)	47 (24.9)
> 45	57 (14.3)	30 (15.0)	27 (13.5)	24 (11.4)	33 (17.5)
CD4 among high CD4 group [median (IQR)] (n = 194)	434.5 (392–481)	434.5 (392–481)	–	449.5 (401–488)	418.5 (380–465)
CD4 among low CD4 group [median (IQR)] (n = 199)	121 (55–160)	–	121 (55–160)	122.5 (64–164.5)	119 (51–159)
Education					
None	23 (5.7)	11 (5.5)	12 (6.0)	17 (8.1)	6 (3.2)
P1–P6/G1–G7	96 (23.9)	49 (24.5)	47 (23.4)	74 (35.1)	22 (11.6)
P7–S6/G8–G12	258 (64.3)	128 (64.0)	130 (64.7)	112 (53.1)	146 (76.8)
> S6/> G12	24 (6.0)	12 (6.0)	12 (6.0)	8 (3.8)	16 (8.4)
Household asset index (n = 382)					
Low	160 (41.9)	85 (45.0)	75 (38.9)	112 (55.5)	48 (26.7)
Middle	143 (37.4)	66 (34.9)	77 (39.9)	42 (20.8)	101 (56.1)
High	79 (20.7)	38 (20.1)	41 (21.2)	48 (23.8)	31 (17.2)
Months since HIV+ diagnosis [median (IQR)] (n = 385)	1.3 (0.4–19.7)	4.3 (0.6–36.0)	0.9 (0.3–3.7)	0.8 (0.1–18.8)	2.0 (0.7–22.3)
Hopkins depression score [mean (SD)]	1.7 (0.6)	1.6 (0.6)	1.7 (0.6)	1.5 (0.6)	1.8 (0.6)
Structural barriers score [mean (SD)]	7.7 (8.0)	8.3 (8.0)	7.1 (7.9)	2.8 (5.0)	13.0 (7.2)
Physical Health Summary Score (PHS) [mean (SD)] (n = 381)	40.5 (6.9)	42.3 (6.8)	38.7 (6.5)	38.6 (5.1)	42.3 (7.9)
Mental Health Summary Score (MHS) [mean (SD)] (n = 381)	41.1 (11.3)	42.4 (11.0)	39.8 (11.4)	44.0 (12.5)	38.1 (9.0)
Currently on TB medication					
Yes	14 (3.5)	1 (0.5)	13 (6.5)	2 (1.0)	12 (6.3)
No	387 (96.5)	199 (99.5)	188 (93.5)	209 (99.1)	178 (93.7)
Stigma: disclosure concerns score [mean (SD)]	1.7 (1.6)	1.7 (1.6)	1.7 (1.5)	1.8 (1.6)	1.6 (1.5)
Stigma: perceived negative attitudes score [mean (SD)]	1.4 (1.2)	1.3 (1.2)	1.5 (1.2)	1.1 (1.1)	1.7 (1.2)
Unhealthy alcohol use					
Self-report (AUDIT-C positive: women: ≥ 3; men: ≥ 4)					
No	322 (80.3)	154 (77.0)	168 (83.6)	189 (89.6)	133 (70.0)
Yes	79 (19.7)	46 (23.0)	33 (16.4)	22 (10.4)	57 (30.0)
PEth (≥ 50 ng/mL)					
No	272 (67.8)	134 (67.0)	138 (68.7)	142 (67.3)	130 (68.4)
Yes	129 (32.2)	66 (33.0)	63 (31.3)	69 (32.7)	60 (31.6)
AUDIT-C and PEth					
AUDIT-C negative and PEth < 50 ng/mL	251 (62.6)	121 (60.5)	130 (64.7)	140 (66.4)	111 (58.4)
AUDIT-C positive and PEth < 50 ng/mL	21 (5.2)	13 (6.5)	8 (4.0)	2 (0.9)	19 (10.0)
AUDIT-C negative and PEth ≥ 50 ng/mL (Underreporting)	71 (17.7)	33 (16.5)	38 (18.9)	49 (23.2)	22 (11.6)
AUDIT-C positive and PEth ≥ 50 ng/mL	58 (14.5)	33 (16.5)	25 (12.4)	20 (9.5)	38 (20.0)

### Prevalence of Unhealthy Alcohol Use

In the overall sample, 32.2% (*n *= 129) had biomarker-measured unhealthy alcohol use (PEth ≥ 50 ng/mL), compared to 19.7% (*n *= 79) per AUDIT-C (see Table 1). Approximately one-third of participants in the low and the high CD4 groups were positive for PEth-measured unhealthy alcohol use (31.3% and 33.0%, respectively). Nearly one-quarter (23.0%) of those in the high CD4 group self-reported unhealthy alcohol use compared to 16.4% in the low CD4 group. In South Africa, the prevalence of unhealthy alcohol use was similar using PEth (31.6%) versus self-report (30.0%), whereas in Uganda, the prevalence of unhealthy alcohol use was 32.7% via PEth, compared to 10.4% via self-report.

### Correlates of Biomarker-Measured Unhealthy Alcohol Use in South Africa

Bivariate and multivariable analyses of PEth-measured unhealthy alcohol use in South Africa are presented in Table 2. CD4 cell count group was not associated with unhealthy alcohol use in South Africa in bivariate or multivariable analyses. Only gender was significantly associated with unhealthy alcohol use, with females having a lower odds of unhealthy alcohol use compared to males in bivariate (odds ratio (OR) 0.29; 95% Confidence Interval (CI) 0.15–0.56) and multivariable analyses (adjusted odds ratio (AOR) 0.25; 95% CI 0.12–0.54). There was not a significant interaction between CD4 group and PHS in relation to unhealthy alcohol use in South Africa (CD4*PHS interaction term *p* value = 0.45; data not shown).

**Table 2:** Bivariate associations, unadjusted and adjusted odds ratios (OR) and 95% confidence intervals (CI) for unhealthy alcohol use (unhealthy alcohol use defined as PEth ≥ 50 ng/mL) at enrollment, among individuals with PEth and AUDIT-C data in South Africa (n = 190)

	No unhealthy alcohol use N (%) or median (IQR)	Unhealthy alcohol use N (%) or median (IQR)	Unadjusted OR (95% CI)	*p-*value	Adjusted OR (95% CI)	*p*-value
Primary predictor variable						
CD4 group				0.83		0.99
Low CD4 group	65 (67.7)	31 (32.3)	1.00		1.00	
High CD4 group	65 (69.2)	29 (30.9)	0.94 (0.51, 1.73)		1.00 (0.46, 2.17)	
Covariates						
Gender				< 0.01		< 0.01
Male	31 (50.0)	31 (50.0)	1.00		1.00	
Female	99 (77.3)	29 (22.7)	0.29 (0.15, 0.56)		0.25 (0.12, 0.54)	
Age				0.52		0.39
≤ 25	21 (80.8)	5 (19.2)	1.00		1.00	
26–35	55 (66.3)	28 (33.7)	2.14 (0.73, 6.27)		1.79 (0.53, 6.05)	
36–45	32 (68.1)	15 (31.9)	1.97 (0.62, 6.23)		1.27 (0.33, 4.80)	
> 45	21 (63.6)	12 (36.4)	2.40 (0.72, 8.02)		3.01 (0.71, 12.78)	
Household asset index				0.33		0.34
Low	36 (75.0)	12 (25.0)	1.00		1.00	
Middle	65 (64.4)	36 (35.6)	1.66 (0.77, 3.59)		1.98 (0.80, 4.90)	
High	23 (74.2)	8 (25.8)	1.04 (0.37, 2.94)		1.68 (0.51, 5.55)	
Physical health summary score (PHS)	41.5 (36.7–46.8)	42.2 (36.7–48.0)	1.01 (0.97, 1.05)	0.57	1.03 (0.98, 1.08)	0.27
Mental health summary score (MHS)	37.0 (32.2–45.4)	37.0 (33.0–43.9)	1.01 (0.98, 1.05)	0.47	1.03 (0.98, 1.08)	0.22
Hopkins depression score	1.7 (1.4–2.2)	1.7 (1.3–2.3)	0.84 (0.48, 1.45)	0.53	1.57 (0.73, 3.37)	0.25
Stigma: disclosure concerns score	2 (0–3)	1 (0–3)	0.88 (0.72, 1.08)	0.22	0.86 (0.66, 1.13)	0.29
Stigma: perceived negative attitudes score	2 (1–3)	2 (0.5–3)	0.93 (0.72, 1.20)	0.58	0.93 (0.68, 1.28)	0.67
Months since HIV+ diagnosis	1.9 (0.7–19.8)	2.3 (0.9–22.3)	1.00 (0.99, 1.01)	0.63	1.00 (0.99, 1.01)	0.85
Study enrollment date (per 30 days from earliest enrollment)	7.5 (4.1–13.3)	8.8 (5.1–14.4)	1.04 (0.98, 1.11)	0.17	1.04 (0.97, 1.12)	0.29
Structural barriers score	13 (8–18)	11 (8–14.5)	0.96 (0.92, 1.00)	0.07	0.96 (0.90, 1.01)	0.12

### Correlates of Biomarker-Measured Unhealthy Alcohol Use in Uganda

Bivariate and multivariable analyses of PEth-measured unhealthy alcohol use in Uganda are presented in Table 3. CD4 cell count group was associated with PEth-measured unhealthy alcohol use in Uganda in the multivariable model (AOR 2.65; 95% CI 1.05–6.72 for individuals in the high versus low CD4 group); gender was associated with unhealthy drinking in bivariate (OR 0.27; 95% CI 0.15–0.49 for females compared to males) and multivariable analyses (AOR 0.16; 95% CI 0.07–0.36 for females compared to males). There was not a significant interaction between CD4 group and PHS in relation to unhealthy alcohol use in Uganda (CD4*PHS interaction term *p* value = 0.47; data not shown).

**Table 3:** Bivariate associations, unadjusted and adjusted odds ratios (OR) and 95% confidence intervals (CI) for unhealthy alcohol use (unhealthy alcohol use defined as PEth ≥ 50 ng/mL) at enrollment, among individuals with PEth and AUDIT-C data in Uganda (n = 211)

	No unhealthy alcohol use N (%) or median (IQR)	Unhealthy alcohol use N (%) or median (IQR)	Unadjusted OR (95% CI)	*p*-value	Adjusted OR (95% CI)	*p*-value
Primary predictor variable						
CD4 group				0.49		0.04
Low CD4 group	73 (69.5)	32 (30.5)	1.00		1.00	
High CD4 group	69 (65.1)	37 (34.9)	1.22 (0.69, 2.18)		2.65 (1.05, 6.72)	
Covariates						
Gender				< 0.01		< 0.01
Male	42 (50.0)	42 (50.0)	1.00		1.00	
Female	100 (78.7)	27 (21.3)	0.27 (0.15, 0.49)		0.16 (0.07, 0.36)	
Age				0.40		0.74
≤ 25	42 (75.0)	14 (25.0)	1.00		1.00	
26–35	62 (67.4)	30 (32.6)	1.45 (0.69, 3.06)		0.87 (0.34, 2.20)	
36–45	23 (59.0)	16 (41.0)	2.09 (0.87, 5.03)		1.46 (0.51, 4.20)	
> 45	15 (62.5)	9 (37.5)	1.80 (0.65, 5.01)		1.10 (0.29, 4.09)	
Household asset index				0.23		0.33
Low	76 (67.9)	36 (32.1)	1.00		1.00	
Middle	23 (54.8)	19 (45.2)	1.74 (0.84, 3.60)		1.85 (0.77, 4.48)	
High	34 (70.8)	14 (29.2)	0.87 (0.42, 1.82)		0.93 (0.38, 2.28)	
Physical health summary score (PHS)	38.9 (35.9–42.6)	38.9 (36.2–41.6)	0.99 (0.93, 1.05)	0.70	1.01 (0.94, 1.09)	0.78
Mental health summary score (MHS)	45.9 (34.8–53.8)	49.3 (37.8–56.6)	1.02 (0.99, 1.05)	0.13	1.01 (0.97, 1.05)	0.64
Hopkins depression score	1.3 (1.1–1.8)	1.3 (1.3–1.6)	0.70 (0.41, 1.20)	0.19	0.95 (0.38, 2.37)	0.91
Stigma: disclosure concerns score	2 (0–3)	2 (0–3)	0.99 (0.82, 1.18)	0.88	0.97 (0.77, 1.22)	0.79
Stigma: perceived negative attitudes score	1 (0–2)	0 (0–2)	0.91 (0.71, 1.18)	0.47	1.06 (0.75, 1.51)	0.73
Months since HIV+ diagnosis	0.6 (0.1–17.9)	1.3 (0.2–21.2)	1.00 (0.99, 1.01)	0.80	1.00 (0.99, 1.01)	0.82
Study enrollment date (per 30 days from earliest enrollment)	8.8 (4.6–11.4)	8.3 (5.3–11.2)	1.00 (0.93, 1.07)	0.98	1.00 (0.90, 1.12)	0.99
Structural barriers score	0 (0–4)	0 (0–3)	0.98 (0.92, 1.04)	0.52	0.98 (0.90, 1.07)	0.69

### Prevalence of Underreporting Alcohol Use

In the overall sample, 17.7% (*n *= 71) of individuals underreported unhealthy alcohol use (i.e., PEth ≥ 50 ng/mL but AUDIT-C negative; see Table 1). In the high CD4 group, the prevalence of underreporting was 16.5%, compared to 18.9% in the low CD4 group (*p *= 0.53). In Uganda, 23.2% underreported alcohol use, compared to 11.6% in South Africa (χ^2^ = 9.30; *p *< 0.01).

### Correlates of Underreporting Alcohol Use in South Africa

As shown in Table 4, CD4 cell count group was not associated with underreporting in South Africa in bivariate or multivariable analyses. Only perceived physical health status and gender were associated with underreporting in South Africa in both bivariate and multivariable analyses. In bivariate analyses, the odds of underreporting decreased with increasing PHS scores (OR 0.92; 95% CI 0.86–0.98 for a one-point increase in PHS), and gender (OR 0.29; 95% CI 0.11–0.71 for females compared to males). In the multivariable model, the adjusted odds of underreporting also decreased with increasing perceived physical health status scores (AOR 0.88; 95% CI 0.80–0.97 for a one-point increase in PHS), and gender (AOR 0.16; 95% CI 0.04–0.60 for females compared to males). There was not a significant interaction between CD4 group and PHS in relation to underreporting of alcohol use in South Africa (CD4*PHS interaction term *p* value = 0.65; data not shown).

**Table 4:** Bivariate associations, unadjusted and adjusted odds ratios (OR) and 95% confidence intervals (CI) for under reporting alcohol use (under report defined as AUDIT-C negative and PEth ≥ 50 ng/mL) at enrollment, among individuals with PEth and AUDIT-C data in South Africa (n = 190)

	No under reported alcohol use N (%) or median (IQR)	Under reported alcohol use N (%) or median (IQR)	Unadjusted OR (95% CI)	*p*-value	Adjusted OR (95% CI)	*p*-value
Primary predictor variable						
CD4 group				0.40		0.61
Low CD4 group	83 (86.5)	13 (13.5)	1.00		1.00	
High CD4 group	85 (90.4)	9 (9.6)	0.68 (0.27, 1.67)		1.41 (0.37, 5.40)	
Covariates						
Gender				< 0.01		< 0.01
Male	49 (79.0)	13 (21.0)	1.00		1.00	
Female	119 (93.0)	9 (7.0)	0.29 (0.11, 0.71)		0.16 (0.04, 0.60)	
Age				0.54		0.24
≤ 25	24 (92.3)	2 (7.7)	1.00		1.00	
26–35	73 (88.0)	10 (12.1)	1.64 (0.34, 8.03)		0.76 (0.11, 5.11)	
36–45	43 (91.5)	4 (8.5)	1.12 (0.19, 6.55)		0.08 (0.00, 1.36)	
> 45	27 (81.8)	6 (18.2)	2.67 (0.49, 14.48)		0.94 (0.11, 8.05)	
Household asset index				0.13		0.28
Low	45 (93.8)	3 (6.3)	1.00		1.00	
Middle	86 (85.2)	15 (14.9)	2.62 (0.72, 9.51)		2.78 (0.60, 12.82)	
High	30 (96.8)	1 (3.2)	0.50 (0.05, 5.04)		0.79 (0.06, 9.69)	
Physical health summary score (PHS)	42.1 (37.1–47.6)	37.2 (31.7–43.3)	0.92 (0.86, 0.98)	< 0.01	0.88 (0.80, 0.97)	0.01
Mental health summary score (MHS)	36.7 (32.3–45.4)	38.4 (34.7–41.8)	1.02 (0.97,1.07)	0.47	0.99 (0.91, 1.06)	0.72
Hopkins depression score	1.7 (1.4–2.3)	1.5 (1.3–1.9)	0.45 (0.18, 1.14)	0.09	0.56 (0.14, 2.27)	0.42
Stigma: disclosure concerns score	1 (0–3)	1.5 (0–3)	0.96 (0.72, 1.28)	0.77	0.79 (0.50, 1.25)	0.32
Stigma: perceived negative attitudes score	2 (1–3)	2 (0–3)	0.97 (0.67, 1.40)	0.87	1.00 (0.58, 1.72)	0.99
Months since HIV+ diagnosis	2.1 (0.7–19.8)	1.4 (0.7–24.3)	1.00 (0.99, 1.01)	0.88	1.00 (0.98, 1.02)	0.98
Study enrollment date (per 30 days from earliest enrollment)	7.6 (4.4–13.7)	8.4 (5.2–13)	1.02 (0.93, 1.11)	0.72	1.04 (0.92, 1.18)	0.54
Structural barriers score	12 (8–18)	13 (10–17)	1.02 (0.96, 1.08)	0.52	1.05 (0.95, 1.16)	0.35

### Correlates of Underreporting Alcohol Use in Uganda

As shown in Table 5, CD4 cell count group was not associated with underreporting in Uganda in bivariate or multivariable analyses. In the bivariate analysis, the odds of underreporting increased with increasing mental health status scores (OR 1.03; 95% CI 1.00–1.06 for a one-point increase in MHS) and decreased for females compared to males (OR 0.32; 95% CI 0.16–0.61). In the multivariable analysis, only gender was significantly associated with underreporting (AOR 0.22; 95% CI 0.09–0.52 for females compared to males). There was not a significant interaction between CD4 group and PHS in relation to underreporting of alcohol use in Uganda (CD4*PHS interaction term *p* value = 0.66; data not shown).

**Table 5:** Bivariate associations, unadjusted and adjusted odds ratios (OR) and 95% confidence intervals (CI) for under reporting alcohol use (under report defined as AUDIT-C negative and PEth ≥ 50 ng/mL) at enrollment, among individuals with PEth and AUDIT-C data in Uganda (n = 211)

	No under reported alcohol use N (%) or median (IQR)	Under reported alcohol use N (%) or median (IQR)	Unadjusted OR (95% CI)	*p*-value	Adjusted OR (95% CI)	*p*-value
Primary predictor variable						
CD4 group				0.84		0.35
Low CD4 group	80 (76.2)	25 (23.8)	1.00		1.00	
High CD4 group	82 (77.4)	24 (22.6)	0.94 (0.49, 1.77)		1.59 (0.60, 4.20)	
Covariates						
Gender				< 0.01		< 0.01
Male	54 (64.3)	30 (35.7)	1.00		1.00	
Female	108 (85.0)	19 (15.0)	0.32 (0.16, 0.61)		0.22 (0.09, 0.52)	
Age				0.31		0.76
≤ 25	48 (85.7)	8 (14.3)	1.00		1.00	
26–35	69 (75.0)	23 (25.0)	2.00 (0.83, 4.85)		1.34 (0.47, 3.81)	
36–45	28 (71.8)	11 (28.2)	2.36 (0.85, 6.56)		1.66 (0.51, 5.39)	
> 45	17 (70.8)	7 (29.2)	2.47 (0.78, 7.84)		1.99 (0.50, 8.00)	
Household asset index				0.19		0.26
Low	85 (75.9)	27 (24.1)	1.00		1.00	
Middle	28 (66.7)	14 (33.3)	1.57 (0.73, 3.41)		1.82 (0.73, 4.54)	
High	40 (83.3)	8 (16.7)	0.63 (0.26, 1.51)		0.73 (0.26, 2.04)	
Physical health summary score (PHS)	38.8 (35.7–42.6)	38.9 (36.2–41.8)	1.01 (0.94, 1.07)	0.83	1.04 (0.96, 1.13)	0.36
Mental health summary score (MHS)	45.7 (34.9–53.8)	51.0 (38.3–56.7)	1.03 (1.00, 1.06)	0.06	1.03 (0.98, 1.07)	0.26
Hopkins depression score	1.3 (1.1–1.8)	1.3 (1.1–1.6)	0.64 (0.34, 1.20)	0.16	1.17 (0.44, 3.13)	0.76
Stigma: disclosure concerns score	2 (0–3)	1 (0–3)	0.88 (0.71, 1.08)	0.21	0.89 (0.69, 1.14)	0.34
Stigma: perceived negative attitudes score	1 (0–2)	0 (0–2)	0.82 (0.61, 1.09)	0.17	1.01 (0.69, 1.48)	0.97
Months since HIV+ diagnosis	0.7 (0.1–17.5)	1.2 (0.2–21.9)	1.00 (0.99, 1.01)	0.70	1.00 (0.99, 1.01)	0.84
Study enrollment date (per 30 days from earliest enrollment)	8.6 (4.7–11.5)	8.4 (5.3–11)	1.01 (0.93, 1.09)	0.86	0.97 (0.86, 1.09)	0.59
Structural barriers score	0 (0–4)	0 (0–2)	0.96 (0.89, 1.03)	0.27	0.97 (0.89, 1.07)	0.58

## Discussion

This study assessed how CD4 count at ART initiation related to biomarker-measured unhealthy alcohol use among individuals living with HIV at two sites in sub-Saharan Africa. As individuals are now initiating ART at all levels of health, it is important to understand the extent to which unhealthy alcohol use may interfere with successful HIV treatment outcomes in healthier populations. Previous research among individuals living with HIV/AIDS in Uganda showed that unhealthy alcohol use at ART initiation was associated with fewer physical health symptoms [10], and similar research in other chronic conditions also has showed that worsening physical health status is associated with reduced alcohol consumption [11, 12]. Thus, we hypothesized that when HIV symptoms were fewer, alcohol use may be more of a problem.

We observed very high rates of unhealthy alcohol use overall. Almost one-third of patients sampled had biomarker-measured levels of alcohol use greater than 50 ng/mL, reflecting a very high rate of unhealthy drinking, whereas only one-fifth self-reported unhealthy alcohol use. Overall, approximately 17.7% of individuals underreported alcohol use (i.e., were PEth positive but AUDIT-C negative), which was higher in Uganda (23.2%) compared to South Africa (11.6%). These findings are consistent with prior research in South Africa that identified 27% of patients as heavy drinkers from hair testing of ethyl glucuronide (EtG), whereas only 15% self-reported heavy alcohol use [13]. Similarly, prior research in Uganda among individuals living with HIV who reported any past year alcohol use found that 36% of individuals had underreported alcohol use (i.e., were PEth positive but AUDIT-C negative) [34]. Identifying who is most likely to have unhealthy alcohol use at ART initiation may inform efforts to provide additional adherence support and counseling for unhealthy alcohol use.

We found that higher CD4 count was associated with biomarker-measured unhealthy alcohol use in Uganda, but not in South Africa. That is, individuals initiating ART at higher CD4 counts when asymptomatic were more likely to have unhealthy alcohol use in Uganda. This finding is consistent with our hypothesis that patients starting ART at higher CD4 counts would be healthier and more interested in drinking alcohol than those with lower CD4 counts, and that as health declines, alcohol becomes less appealing. This trend is supported by prior research in HIV showing that unhealthy alcohol use at ART initiation was associated with fewer physical health symptoms [10], as well as the “sick quitter” hypothesis—which has shown that as physical health declines, individuals are more likely to be abstinent from alcohol [12]. It is unclear why higher CD4 count was associated with unhealthy alcohol use in Uganda but not in South Africa. South Africa has a higher prevalence of heavy episodic drinking in the general population (10.4%) compared to Uganda (3.4%) [35, 36]. Potential factors to explain this difference may arise from social norms related to drinking alcohol [37–39] and/or cost of alcohol in the context of distinct country income levels [40]. However, future comparative work is necessary to fully understand this finding. Regardless, almost one-third of patients drinking at unhealthy levels when starting ART is problematic, and the level of underreporting, especially in Uganda, makes this an even more challenging issue.

Further, individuals in the high CD4 group who were asymptomatic perceived themselves as healthier than individuals in the low CD4 group. However, there was no association between *perceived* health status and unhealthy drinking in either site, nor an interaction between CD4 count and perceived physical health status in relation to unhealthy drinking at either site. These findings suggest that health status may be experienced or perceived in different ways, which may then be associated with other behaviors, such as alcohol use. More attention to these issues will be needed as public policy encourages initiation of ART among increasing numbers of individuals with high CD4 counts.

In both sites, males were more likely to have biomarker-measured unhealthy alcohol use compared to females, consistent with prior literature demonstrating that males are more likely to drink unhealthily compared to females [41]. This finding also reflects social norms around drinking among men versus women even in the absence of HIV infection. Many societies hold more negative attitudes towards women’s drinking alcohol than men’s drinking, and especially towards their harmful drinking [42–44]. We also found gender differences in underreporting in both sites. Females were significantly less likely to underreport alcohol use compared to males in Uganda and South Africa, which was expected given their lower consumption overall compared to men based on PEth results.

Comparing biomarker-measured and self-reported unhealthy alcohol use in Uganda compared to South Africa, we found significantly higher rates of underreporting of alcohol use in Uganda compared to South Africa. South Africa had much higher self-reported unhealthy alcohol on the AUDIT-C compared to Uganda, yet there were similar rates of biomarker-measured alcohol use across sites. This finding may reflect different social norms around alcohol use in the two settings, including influences from religion [45], degree of stigma [46], social desirability biases [47], and desire to please health care workers [48, 49]. Further comparison of these factors may help explain the different rates of underreporting we observed across sites. Future work is needed, particularly in Uganda, to identify strategies to screen for and treat unhealthy alcohol use in the context of high underreporting.

However, screening and treating alcohol use in HIV care in Uganda may be challenging, given that our findings showed no factors were significantly associated with underreporting in Uganda (with the exception of gender, which reflects known gender differences in alcohol consumption). In South Africa, only self-reported physical health status was associated with underreporting; individuals in South Africa who perceived themselves as healthier were more likely to report alcohol use, and individuals who perceived themselves as sicker were more likely to minimize their alcohol use. Yet, actual immunological status, as measured by CD4 count group, was unrelated to underreporting in South Africa or Uganda. Further, the interaction between one’s *perception* of their health status and their immunological status was not related underreporting at either site.

Our results must be interpreted in light of study limitations, including the use of a cross-sectional design, which limits our ability to interpret the directionality of findings. Further, the rates of unhealthy alcohol use and underreporting may differ significantly by community and across distinct demographic groups; this is reflected in other work evaluating PEth in diverse samples, including HIV-infected young women in Russia [50], young people who inject drugs in the US [51], and veterans living with HIV and/or hepatitis C [52]. As such, these findings may not generalize to other populations. Despite these limitations, these findings suggest that unhealthy alcohol use is quite common among individuals initiating ART in both settings and may be a barrier to successful treatment outcomes.

## Conclusions

These findings have important clinical implications in South Africa and Uganda. Biomarker-measured unhealthy alcohol use was prevalent in both Uganda and South Africa, supporting the need for alcohol use interventions to be integrated into HIV care. Innovative methods for screening for alcohol use in the context of under reporting are needed. At the time of this study, PEth is only being used as a research tool, as cost and access to a specialty laboratory are currently barriers to incorporating it into routine care. Future work is needed to identify affordable, rapid tests for alcohol biomarkers to detect alcohol use early in HIV treatment, for instance point-of-care testing for alcohol biomarkers such as urine EtG [53], especially in settings such as Uganda where underreporting is high. Further, in expanding efforts to screen and treat unhealthy alcohol use in HIV care, it will be important in future work to consider how to tailor interventions for people who are healthier and may be less motivated to reduce drinking.
